# *Bacillus cereus *cytotoxins Hbl, Nhe and CytK are secreted via the Sec translocation pathway

**DOI:** 10.1186/1471-2180-10-304

**Published:** 2010-11-30

**Authors:** Annette Fagerlund, Toril Lindbäck, Per Einar Granum

**Affiliations:** 1 Norwegian School of Veterinary Science, Department of Food Safety and Infection Biology, PO Box 8146 Dep., N-0033 Oslo, Norway; 2 Laboratory for Microbial Dynamics (LaMDa) and Department of Pharmaceutical Biosciences, University of Oslo, PO Box 1068 Blindern, N-0316 Oslo, Norway

## Abstract

**Background:**

*Bacillus cereus *and the closely related *Bacillus thuringiensis *are Gram positive opportunistic pathogens that may cause food poisoning, and the three secreted pore-forming cytotoxins Hbl, Nhe and CytK have been implicated as the causative agents of diarrhoeal disease. It has been proposed that the Hbl toxin is secreted using the flagellar export apparatus (FEA) despite the presence of Sec-type signal peptides. As protein secretion is of key importance in virulence of a microorganism, the mechanisms by which these toxins are secreted were further investigated.

**Results:**

Sec-type signal peptides were identified in all toxin components, and secretion of Hbl component B was shown to be dependent on an intact Sec-type signal peptide sequence. Further indication that secretion of Hbl, Nhe and CytK is dependent on the Sec translocation pathway, the main pathway on which bacterial secretion relies, was suggested by the observed intracellular accumulation and reduced secretion of the toxins in cultures supplemented with the SecA inhibitor sodium azide. Although a FEA deficient strain (a *flhA *mutant) showed reduced toxin expression and reduced cytotoxicity, it readily secreted overexpressed Hbl B, showing that the FEA is not required for Hbl secretion. Thus, the concurrent lack of flagella and reduced toxin secretion in the FEA deficient strain may point towards the presence of a regulatory link between motility and virulence genes, rather than FEA-dependent toxin secretion.

**Conclusions:**

The Hbl, Nhe and CytK toxins appear to be secreted using the Sec pathway, and the reduced Hbl expression of a FEA deficient strain was shown not to be due to a secretion defect.

## Background

*Bacillus cereus *and the closely related *Bacillus thuringiensis *are Gram positive bacteria belonging to the *B. cereus *group, recognized as causative agents of gastrointestinal disease. Three pore-forming toxins appear to be responsible for the diarrhoeal type of food poisoning: Hemolysin BL (Hbl), Non-haemolytic enterotoxin (Nhe), and Cytotoxin K (CytK) [1]. Since *B. thuringiensis *is only differentiated from *B. cereus *by the presence of plasmids encoding insecticidal crystal toxins [2], *B. cereus *and *B. thuringiensis *show a similar prevalence and expression of genes encoding these cytotoxins [3,4]. Hbl and Nhe each consist of three different protein components, named L_2_, L_1_, and B, and NheA, NheB and NheC, respectively, while CytK is a single-component toxin [1].

The expression of the *B. cereus *cytotoxins is positively regulated by a quorum sensing system composed of the transcriptional activator PlcR and its activating peptide PapR [5]. Expression of Hbl and Nhe is also regulated by the redox-sensitive two-component regulatory system ResDE and the redox regulator Fnr [6,7], and to a lesser extent the catabolite control protein CcpA [8], demonstrating a link between virulence and the metabolic state of the cell. In many pathogenic bacteria, the expression of motility genes and virulence factors are co-ordinately regulated [9], and a regulatory link between motility and virulence appears to exist also in *B*. *cereus *and *B. thuringiensis*, which are motile by peritrichous flagella. For example, motility was reduced in a *plcR *mutant [10], transcription of the genes encoding Hbl and phosphatidylinositol-specific phospholipase C was reduced in the non-flagellated *flhA *mutant [11], and Hbl production increased during swarming migration [12]. However, the molecular mechanisms that putatively couple the expression of virulence factors to motility have not been elucidated.

Protein secretion is of key importance in virulence of a microorganism, as bacterial protein toxins must cross the bacterial membrane(s) in order to gain access to their site of action at the target host cell. It has been suggested that the Hbl proteins are secreted using the flagellar export apparatus (FEA), as non-flagellated strains were deficient in Hbl secretion [12,13], but the pathways used to translocate Nhe and CytK from the bacterial cell have not been investigated. In Gram positive bacteria, in which secreted proteins only have to cross a single lipid bilayer, six protein secretion systems are currently recognized [14-16]: The general secretory (Sec) pathway, the twin arginine targeting (Tat) pathway, the fimbrillin-protein exporter (FPE), the FEA, the holins, and the WXG100 secretion system (Wss). The Sec pathway is considered the general housekeeping protein translocation system and is essential in all bacteria for which it has been studied. To gain further insight into the pathogenesis of *B*. *cereus *and the relationship between toxin production and motility in this bacterium, the current study aims to elucidate which secretion pathway is used to export the *B. cereus *Hbl, Nhe and CytK cytotoxin components.

## Results and discussion

### The *B. cereus *cytotoxins contain Sec-type signal peptide sequences

**Figure 1:**
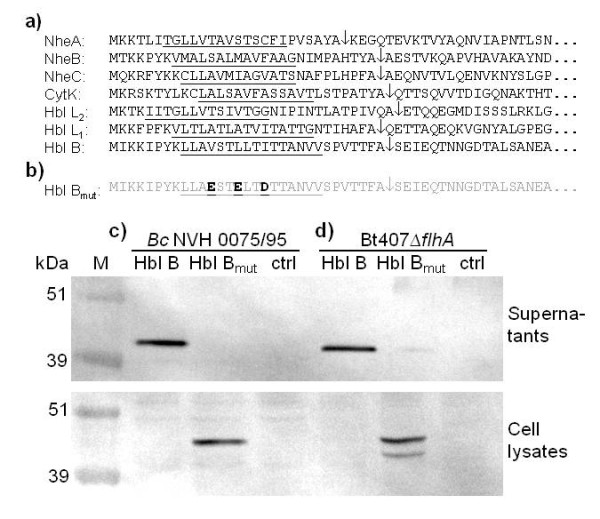
**The *B. cereus *toxins contain Sec-type signal peptides**. (**A**) Sec-type signal peptide sequences of the Hbl, Nhe and CytK cytotoxin proteins from *B. cereus *ATCC 14579 predicted using SignalP. The predicted cleavage sites are marked with arrows and the hydrophobic regions are underlined. (**B**) Site-directed mutations introduced into the hydrophobic core of the signal peptide of Hbl B in this study. Western immunoblot analysis of Hbl B in culture supernatants and cell lysates of (**C**) *B. cereus *(*Bc*) NVH0075/95 and (**D**) the non-flagellated *B. thuringiensis *407 *flhA *mutant (Bt407Δ*flhA*) transformed with pHT304-pXyl expressing native Hbl B and Hbl B with a mutated signal peptide sequence (Hbl B_mut_). Negative controls are strains harbouring pHT304-pXyl empty vector (ctrl). Expression was induced by 20 mM xylose. Lane M: molecular weight marker.

Sec-type signal peptides target proteins for secretion via the Sec translocation pathway, and are characterized by a positively charged amino-terminus, a stretch of hydrophobic residues and a cleavage site for a signal peptidase [17,18]. The protein components of the *B. cereus *toxins Hbl, Nhe, and CytK all contain Sec-type signal peptides, as determined by analysis using the SignalP prediction method [19] (Figure 1A).

Signal peptides are cleaved upon secretion. In the original reports describing Hbl, Nhe, and CytK, amino-terminal sequencing using Edman degradation was performed on proteins purified from culture supernatants. These sequences correspond to the predicted amino-termini of the mature proteins in the case of all three Hbl proteins, NheB and CytK [20-22]. The amino-terminal sequence of purified NheA started 11 amino acids downstream of the predicted signal peptidase cleavage site [21], but since a slightly larger form of NheA has also been isolated [23], this protein probably represents a further processed form. NheC has not been purified from culture supernatant and thus has not been subjected to amino-terminal sequencing. Secretion of CytK into the periplasmic space in the Gram negative *Escherichia coli *[24] further indicates that CytK is produced with a functional signal peptide.

To examine whether the signal peptide sequence was required for secretion of one of the Hbl components, the gene encoding Hbl B was expressed from the *xylA *promoter on a low-copy plasmid. Three of the uncharged amino acid residues present in the hydrophobic core of the Hbl B signal peptide were replaced with negatively charged, hydrophilic amino acid residues: V12E, L15E and I18 D (Figure 1B). Hbl B with intact and mutant signal peptides were expressed in the Hbl-negative strain *B. cereus *NVH 0075/95, and the levels of expressed protein in the supernatant and cell lysate was examined using Western blot analysis (Figure 1C). The results show that Hbl B with intact signal peptide was secreted into the culture supernatant, while Hbl B containing the mutant signal peptide was exclusively associated with the cell pellet, confirming that secretion of Hbl B was dependent on an intact signal peptide sequence.

### Hbl B secretion is not dependent on the FEA

The components of the flagellar export apparatus (FEA) are homologous to the proteins of type III secretion systems present in many Gram negative bacteria [25,26], and exports flagellar proteins into the central channel found within the flagellar basal body complex. It has been claimed that the FEA is required for Hbl secretion, as three non-flagellated *B*. *cereus*/*B. thuringiensis *strains were shown to fail to secrete Hbl [12,13]. However, it was not determined whether the reduction in the level of secreted Hbl was due to reduced transcription, translation, or a secretion defect. To further investigate the secretion pathway of Hbl, Hbl B with intact and mutant signal peptides were expressed as described above in one of the previously described *B. thuringiensis *non-flagellated strains, Bt407 mutated in *flhA *encoding a component of the FEA [13] (Figure 1D). This approach clearly showed that overexpressed Hbl B was secreted in the FEA deficient strain, demonstrating that the FEA was not required for secretion of Hbl B. Overexpressed Hbl B with mutant signal peptide was almost exclusively associated with the cell pellet. A second band of lower molecular weight than intact Hbl B in the lane containing the cell pellet from the FEA-deficient strain likely represents a degradation product of mutant Hbl B, while a weak band in the lane containing the supernatant fraction may represent native chromosomally encoded Hbl B protein or originate from lysed cells.

### Secretion of cytotoxins was inhibited by the SecA inhibitor azide

The Sec translocation pathway in Gram positive bacteria is composed of the SecYEG membrane channel and of SecA, the ATPase that drives the translocation reaction through the SecYEG channel. Sodium azide markedly inhibits Sec-dependent preprotein membrane translocation *in vivo *and *in vitro *[27]. Although azide also inhibits other ATPases [28], it has been shown both in *E. coli *and in *Bacillus subtilis *that azide-resistance may be conferred by specifically mutating SecA [29-31], indicating that SecA is the major target for the lethal action of azide in bacteria. Since deletion mutants in essential components of the Sec translocation pathway are non-viable [32], the Sec-dependence of *B. cereus *Hbl, Nhe, and CytK toxin secretion was investigated by addition of sodium azide to cultures of *B. cereus *ATCC 14579. For this purpose, it was essential to study the secretion of *de novo *synthesised toxins, otherwise the effect of azide would be overshadowed by toxins accumulated in the growth medium. Therefore, cells grown to transition phase (t_0_) were washed and resuspended in culture medium with and without added azide. Culture supernatants were harvested 20 minutes after addition of azide, to minimize pleiotropic effects potentially affecting toxin secretion indirectly. Furthermore, activation of PlcR, the transcriptional regulator required for *B*. *cereus *cytotoxin expression, is dependent on PapR, a 48 amino acid peptide with a Sec-type signal peptide thought to be secreted by the Sec pathway and reimported after extracellular processing [33]. To ensure that potential inhibition of toxin secretion by addition of azide was not an indirect effect due to lack of PapR secretion, a culture containing both azide and synthetic PapR pentapeptide was included. The concentration of azide used (2 mM) was chosen as this was the lowest concentration of azide that inhibited growth of *B. cereus *ATCC 14579 on agar plates.

**Figure 2:**
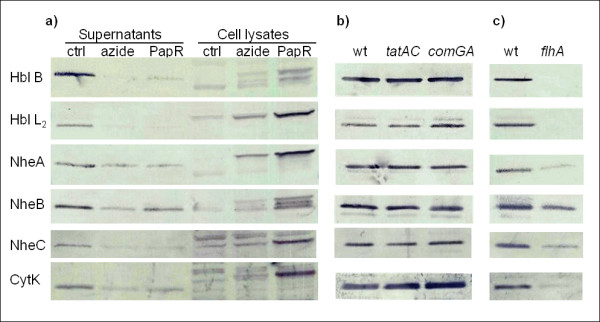
**Western immunoblot analysis of the level of toxin components upon treatment with the SecA inhibitor azide and in Tat, Com, and FEA mutants**. (**A**) Western blots showing the level of toxin components in *B. cereus *ATCC 14579 culture supernatants and cell lysates harvested 20 minutes after cells grown to transition phase were washed and resuspended in fresh culture medium with 2 mM sodium azide (azide) or 2 mM sodium azide and 200 μM PapR pentapetide (PapR). The control culture (ctrl) was grown in BHI only. Toxin components in culture supernatants from (**B**) *B. cereus *ATCC 14579 wild-type (wt), Δ*tatAC*, and Δ*comGA *strains (**C**) *B. thuringiensis *407 (wt) and its non-flagellated *flhA *mutant, harvested one hour into stationary phase.

The Western blot analysis shown in Figure 2A detecting Hbl, Nhe, and CytK proteins shows that in the presence of azide, secretion of the toxins into the culture medium was reduced, while cell lysates contained increased levels of toxins, indicating intracellular accumulation. Incomplete inhibition of toxin secretion in the presence of azide may be due to residual activity of the SecA ATPase at the azide concentration employed. Multiple band patterns in the cell lysates are likely to represent pre-proteins, mature forms, and/or intracellularly degraded forms of the toxins. Addition of PapR to the azide-containing culture resulted in increased toxin production, as cell lysates contained increased levels of the examined toxin proteins, and is probably the result of enhanced activation of PlcR. Importantly, this increase was only observed in the intracellular fraction, and addition of PapR did not alleviate the reduction in the amount of toxins secreted into the culture medium caused by the addition of azide. The effect of azide on secretion of Hbl component L_1 _could not be assessed, as we were unable to detect this component in culture supernatants of the wild-type strain, probably as this protein was only produced in detectable amounts at a time-point later in the growth phase [34]. The toxicity of culture supernatants was measured using the Vero cell cytotoxicity assay [35], showing that addition of azide to the culture reduced supernatant cytotoxicity fivefold (Table 1). These results, together with the detection of Sec-type signal peptides and the demonstration that the signal peptide of Hbl B was essential for secretion, indicate that Hbl, Nhe, and CytK secretion is mediated through the Sec translocation pathway.

**Table 1:** Percentage inhibition of protein synthesis in Vero cells upon addition of varying volumes of concentrated culture supernatants.

Strains and samples	Supernatant concentration factor	Amount of added concentrated supernatant						Volume for 50% inhibition*
			
		0.3 μl	1 μl	3 μl	10 μl	30 μl	100 μl	
ATCC 14579 without azide	40-fold	-4%	21%	37%	89%			4.0 μl
ATCC 14579 with azide	40-fold			-7%	9%	70%	100%	20 μl

ATCC 14579	ten-fold	-2%	50%	97%	100%			1.0 μl
ATCC 14579 Δ*tatAC*	ten-fold	2%	45%	99%	100%			1.1 μl
ATCC 14579 Δ*comGA*	ten-fold	-5%	49%	99%	100%			1.0 μl

Bt407 [*plcA'Z*]	ten-fold	-2%	44%	90%	100%			1.2 μl
Bt407 [*plcA'Z*] Δ*flhA*	ten-fold			16%	72%	100%	100%	6.0 μl

*Amount of supernatant required for 50% inhibition of protein synthesis (measured by C^14^-leucine incorporation) in Vero cells [35].

### Other secretion pathways do not appear to be involved in toxin secretion

In addition to the Sec pathway and the FEA, four other protein secretion systems are currently recognized in Gram positive bacteria [14]. Analysis of the *B. cereus *genome sequences showed that *B. cereus *encodes the minimal Tat transport machinery [36] and homologues to the FPE, which is the dedicated pseudopilin export pathway and part of the competence development pathway [14,37]. Analysis of mutants in *tatAC*, encoding the Tat machinery, and *comGA*, encoding an essential component of the FPE, showed that these pathways were not required for secretion of Hbl, Nhe, and CytK (Figure 2B; Table 1).

Gram positive bacteria furthermore have two specialized secretion systems: holins secreting murein hydrolases [38] and the WXG100 secretion system secreting WXG100 (ESAT-6) family proteins [39,40]. Since the B. cereus Hbl, Nhe, or CytK proteins show no resemblance to murein hydrolases or the WXG100 family of proteins it is unlikely that they are exported using these specialized secretion systems, although it cannot be absolutely excluded from our experiments.

### The *flhA *mutant shows reduced toxin expression and reduced cytotoxicity

It was established above, using an overexpression system, that secretion of Hbl B was not dependent on the FEA (Figure 1D). Further investigation of the Bt407 FEA deficient *flhA *mutant by Western immunoblotting (Figure 2C) and Vero cell cytotoxicity assays (Table 1) clearly showed that the culture supernatant contained reduced amounts of the toxin components compared to the wild-type strain. This reduction could not be alleviated by addition of 200 μM synthetic PapR pentapeptide to cultures of the *flhA *mutant strains (results not shown). The absence of detectable amounts of Hbl L_2 _or B proteins secreted from the *flhA *mutant (Figure 2C) confirms the previous lack of detection of Hbl proteins in culture supernatant from this strain [13]. In contrast, the observed reduced levels of CytK in Δ*flhA *culture supernatant contrasts with the previous lack of detection of reduced CytK (HlyIV) production by the *flhA *mutant in a blood overlay assay [13]. This discrepancy may however be due to the greater sensitivity of the currently used technique. Importantly, no intracellular accumulation of any of the Hbl, Nhe, or CytK toxin components were detected in cell lysates from the *flhA *mutant using Western blot analysis (results not shown), in contrast to the intracellular accumulation of toxins observed in the cell lysates in the azide-treated cultures (Figure 2A) and in cell lysates from the strains overexpressing Hbl B with mutant signal peptide; Hbl B_mut _(Figure 1C and 1D). In the case of Hbl B expression in the flhA mutant, our result contrast with that of a previous report [13] in which an intracellular protein interpreted to be a degraded form of Hbl B was detected, indicating either that the monoclonal antibody employed in that report cross-reacted with a different protein or that the epitope detected by the monoclonal antibody 2A3 against component B [41] used in the current study was not present. Although the growth rate at 32°C was somewhat lower for the *flhA *mutant compared with the parent strain, this was not the reason behind the reduced cytotoxicity and toxin expression of the *flhA *mutant, as both strains reached the same cell number at the time of harvest.

### Potential for coordinated regulation of motility and virulence gene expression

Given the data presented in the current study, the concurrent lack of flagella and reduced toxin secretion in the *flhA *mutant is more consistent with a hypothesis of coordinated regulation of motility and virulence genes, rather than FEA-dependent toxin secretion. This is also supported by the previously observed two-fold reduction in transcription of the genes encoding Hbl in the *flhA *mutant [11].

Coordinated regulation of motility and virulence genes has been demonstrated in several pathogenic bacteria (for reviews see e.g. [9,42-44]). While diarrhoea due to *B. cereus *infection presumably occur through destruction of epithelial cells by enterotoxins produced in the small intestine [45,46], the role of motility, if any, in *B. cereus *infection has not been investigated. Nevertheless, several studies suggest that a connection exists between expression of motility and virulence genes also in *B. cereus *and *B. thuringiensis*: First, an avirulent and non-flagellated *B. thuringiensis *mutant (Bt1302) showed greatly reduced phospholipase and haemolytic activity [47]. A spontaneous suppressor mutation was able to reverse these phenotypes, and although motility was only partially restored, this indicated that these unidentified mutations affected a regulatory pathway shared between genes encoding flagellin, phospholipases, and haemolysins [47]. Bt1302 is not likely to be a *flhA *mutant, since their phenotypes differ, for example in expression of flagellin and growth rate at 37°C [11,13,47]. Second, PlcR, the transcriptional activator of *B. cereus *extracellular virulence factors, appears to also affect motility, as a *plcR *mutant showed reduced motility on agar plates and reduced flagellin expression [10,48]. Third, Hbl production was shown to increase during swarming migration [12,49], a differentiated state where elongated and hyperflagellate swarm cells collectively move across solid surfaces [50]. Notably, it was shown that *hbl *genes were upregulated during swarming, concomitant with increased expression of flagellar genes, while the majority of other genes regulated by PlcR, including *plcR*, *nhe*, and *cytK*, were downregulated during swarming [49]. Interestingly, upregulation of the *hbl *operon concomitantly with downregulation of *plcR*, *nhe *and other PlcR-regulated genes was also observed in a deletion mutant of the two-component system *yvfTU *[51]. Finally, the non-flagellated *B. thuringiensis flhA *mutant examined in the current study additionally shows: (i) reduced phospholipase C activity towards phosphatidylcholine in a gel diffusion assay [13], (ii) two-fold reduction in transcription of the genes encoding Hbl and phosphatidylinositol-specific phospholipase C, (iii) a generally lower (twofold) protein concentration in culture supernatants, (iv) reduced sporulation efficiency and ampicillin resistance [11], (v) reduced bacterial adhesion to eukaryotic cells [52], (vi) and reduced growth rate at 32°C. FlhA from *B*. *subtilis *was shown to act as an adaptor that interacted with the flagella building blocks flagellin and filament-capping protein FliD, and coordinated their delivery to the FEA [53].

The fact that the *B. thuringiensis flhA *mutation is pleiotropic supports the hypothesis that regulatory pathways are affected, although further work is required to elucidate the molecular mechanisms linking the flagellar assembly defect and the pleiotropic nature of the *flhA *mutant. The failure of exogenously added PapR to restore toxin production in the *flhA *mutant indicates that the relationship between the flagellar assembly defect and toxin expression may be complex.

In contrast to most bacterial systems where a hierarchical regulatory cascade controls the temporal expression and production of flagella, regulation of flagellar motility genes appear to be nonhierarchal in *B. cereus *group bacteria [13], similar to the situation in *Listeria monocytogenes*, in which flagellar motility is regulated by the transcriptional repressor MogR [54,55]. Genes encoding MogR are only found in *Listeria *and *B. cereus *group species. Interestingly, when allowing one mismatch to the *L. monocytogenes *consensus MogR site [56], four putative MogR binding sites are found in the *hbl *promoter. However, further work is required to determine whether a regulatory link between *hbl *and motility gene expression in *B. cereus *group bacteria may involve MogR.

## Conclusions

The Hbl, Nhe and CytK toxins appear to be secreted using the Sec pathway, as suggested by reduced secretion and intracellular accumulation of these toxins in cultures supplemented with the SecA inhibitor azide and by the presence of Sec-type signal peptides, which for Hbl B was shown to be required for toxin secretion. The previous suggestion of FEA dependent Hbl secretion [12,13] was not supported by results from the current study, since secretion of Hbl B was shown to be independent of the FEA. Instead, the reduced toxin production exhibited by the FEA deficient mutant potentially points towards unidentified regulatory links between motility and virulence gene expression in *B. cereus *group bacteria.

## Methods

### Bacterial strains

*B. cereus *strain ATCC 14579 was used for assessing the effect of azide on toxin secretion, for creation of deletion mutants, and for PCR-amplification of *hblA*. *B. cereus *NVH 0075/95 [21], lacking genes encoding Hbl [57], was used for overexpression of Hbl component B with and without intact signal peptide sequence. The acrystalliferous *B. thuringiensis *407 Cry^- ^[*plcA'Z*] (Bt407) [58] and its nonmotile *flhA *null mutant MP02 [13], were kind gifts from Dr Emilia Ghelardi (Universita degli Studi di Pisa, Italy). These strains are indistinguishable from the *B. cereus *species due to loss of the plasmids encoding insecticidal crystal toxins [2,59].

### Construction of overexpressing strains and deletion mutants

The low-copy number *E. coli*/*Bacillus *shuttle vector pHT304-pXyl, in which *xylR *and the *xylA *promoter from *Bacillus subtilis *was inserted into the pHT304 cloning site [60] allowing xylose-inducible expression of downstream cloned genes, was a kind gift from Dr Didier Lereclus (INRA, France). The gene encoding Hbl B, *hblA *[61], was PCR amplified from *B*. *cereus *ATCC 14579 using primers tatggatcctaaattggaggaaaatgaaatg and tagaggtaccatgttttagttcactttacaa and inserted into pHT304-pXyl using the primer-incorporated BamHI and KpnI restriction sites (underlined). The resulting plasmid was subjected to site directed mutagenesis using the QuikChange mutagenesis protocol (Stratagene) in order to express a mutated form of Hbl B in which three of the amino acids in the hydrophobic central section of the signal peptide sequence were changed into negatively charged amino acids (Figure 1A and 1B). The plasmids were introduced by electroporation into *B. cereus *NVH 0075/95 and Bt407Δ*flhA *[62].

The *tatAC *operon and the *comGA *gene in *B. cereus *ATCC 14579 were deleted by allelic exchange with a spectinomycin resistance cassette (Sp^R^) from pDG1726 [63] as described [64].

### Growth conditions and sample preparation

*B. cereus *and *B. thuringiensis *were grown in brain heart infusion (BHI) medium at a temperature of 32°C, since toxin production generally is maximal at this temperature [65]. For analysis of Hbl B overexpressing strains, strains containing plasmid were grown for 3 hours in BHI supplemented with 10 μg ml^-1 ^erythromycin, induced with 20 mM xylose and grown for 2 hours before harvesting. For analysis of mutant strains, overnight cultures were supplemented with 250 μg ml^-1 ^spectinomycin, and culture supernatants and pellets were harvested 1 hour after the onset of stationary phase (t_0_), as the concentration of toxins appears to be maximal at this time [34]. t_0 _was defined as the breakpoint in the slope of the vegetative growth phase curve as determined by measuring the optical density at 600 nm. For analysis of inhibition of SecA by sodium azide, ATCC 14579 was grown to t_0_, washed twice in pre-warmed BHI, and resuspended in the original volume of fresh pre-warmed BHI. The culture was divided into three cultures: one containing BHI only, one containing 2 mM sodium azide, and one containing 2 mM sodium azide and 200 μM synthetic PapR pentapeptide LPFEY (corresponding to the five carboxy-terminal amino acids in PapR from *B. cereus *ATCC 14579), incubated as before for a further 20 minutes, and harvested by centrifugation. Culture supernatants were collected by centrifugation and concentrated tenfold for examination of Hbl B overexpressing strains and the *tatAC*, *comGA*, and *flhA *mutants, or 40-fold for azide-treated cultures, by precipitation with 80% ammonium sulphate. Precipitated proteins were dissolved in and dialysed against TES buffer (20 mM Tris pH 7.5, 0.8% NaCl, 1 mM EDTA). For Western blot analysis of cultures with and without azide and PapR, supernatant proteins were concentrated 40-fold by precipitation with four volumes of ice-cold acid acetone:methanol (1:1 v/v), stored at -20°C overnight, harvested by centrifugation and resuspended in TES buffer. For preparation of cell lysates, cells were washed once with cold PBS buffer, resuspended in TES buffer to 10% of the original volume of culture. For Hbl B overexpressing strains, cells were lysed by mechanical disruption using Lysing Matrix B (MP Biomedicals) in a Mini-BeadBeater-8 (BioSpec) according to manufacturer's specifications. For mutant strains and azide-treated cultures, cells were lysed by incubation at 37°C for 60 minutes with 1 mg ml^-1 ^lysozyme, followed by six rounds of freezing and thawing. All samples were used within 2 weeks and all experiments were performed at least twice.

### Analysis of samples

Protein electrophoresis was performed using the NuPAGE Novex Bis-Tris gel systems (Invitrogen), using the SeeBlue Plus2 Pre-Stained Standard (Invitrogen) as the molecular weight marker. Western blot analysis was performed according to standard protocols [66]. Monoclonal antibodies 8B12 against Hbl L_2_, 2A3 and 1B8 against Hbl B, and 1C2 against NheB and Hbl L_1_, 1A8 against NheA (all diluted 1:15), and rabbit antiserum against NheC diluted 1:2000 [41,67,68] were a kind gift from Dr Erwin Märtlbauer (Ludwig-Maximilians-Universität, Munich, Germany). For detection of CytK, rabbit antiserum diluted 1:2000 was used [24].

The Vero cell cytotoxicity assay was performed as described [35] and measures the percentage inhibition of C^14^-leucine incorporation in cells due to the cells being subjected to toxins, calculated relative to a negative control where cells were not subjected to toxin sample. The experiments were performed twice, with two to four parallels in each experiment.

## Authors' contributions

AF participated in the study design, constructed plasmids and mutants, performed cytotoxicity assays, and wrote the manuscript. AF and TL performed transformations, sampling and Western blot analysis, and TL participated in writing of the manuscript. PEG conceived of the study, participated in its design, and critically revised the manuscript. All authors read and approved the final manuscript.
